# On the Concrete Oil Found in the Blood

**Published:** 1831-01-01

**Authors:** 


### XXXVI.

### AN ACCOUNT OF A CONCRETE OIL EXISTING AS A CONSTITUENT PRINCIPLE OF HEALTHY BLOOD. By B. G. BABINGTON, M.D. F.R.S.

IN the recent volume of the Medico-Chirurgical Transactions, this paper of Dr. Babington's is printed. It appears that the only method by which this concrete oil was separated from the blood was by agitation with æther—though from milky serum, in which the said oil abounds, it may be obtained by means of alcohol, or even by simple evaporation.

"This oil is obtained from the serum of healthy blood, whether that serum be milky, opalescent, or clear, and appears to be the chief, if not the only cause, of the colour of healthy serum.

It is of a deep yellow hue, is semi-solid, and melts at a temperature of 90° Fahrenheit. It is lighter than water, its specific gravity being ·918. From its solution in æther, it crystallizes by very slow evaporation, at a low temperature, in radiated tufts. It burns with a brilliant light, has a faint and peculiar odour, resembling that of a wet bladder, forms soaps with the alkalis, and in its general characters resembles other animal oils. It is uniform in colour, in general appearance, and in all its properties, from whatever kind of serum it be obtained."

About one-third part of æther should be added to the serum, in a well-corked phial, which should be reversed a few times, without violent agitation. This movement is to be repeated twice or thrice, at intervals of a day or two; when, after a final rest of some hours, the æther will be found to have risen to the top of the liquid, and to have acquired by impregnation with oil, a yellow colour of more or less depth, according to the proportion of oil which the serum may have contained. By means of a glass syringe, the æthereal solution may be collected from the surface of the serum, and, on evaporation, will yield the material in question, together with a small quantity of albumen, which the æther had taken up, and which may be easily separated by a heat sufficient to coagulate it.

Hewson came to nearly the same conclusion as Dr. B. respecting the milky serum of blood—a phenomenon which has been attributed to chyle, and has puzzled physicians to explain it. Dr. Christison still more recently employed æther as a solvent of the oil in question; so that Dr. Babington has been anticipated in his discovery, though he does not appear to have been acquainted with Dr. Christison's experiments when his own were made.

While analyzing Dr. Babington's paper, we received the following case from Dr. Wilson at Rio Janeiro. It evidently bears on the present subject.

### CASE OF MILKY FLUID DISCHARGED FROM THE SCROTUM. By JOHN WILSON, M.D. of His Majesty's Ship Warspite.

"Some account of the following case will, I think, be interesting, because it is, in many respects, singular in itself; and because it is very similar to one noticed in the Medico-Chirurgical Review for April, 1830, communicated by Mr. Maxwell to Dr. Titley.

*Case.* Senhor ———, a respectable merchant, in Rio Janeiro, is about 40 years of age, and in the enjoyment of good general health. Five years ago he had an acute inflammatory affection of the scrotum, attended by sharp fever; there was at the time much tumefaction, and severe pain; but it is not known whether the testicles were implicated in the disease. At the end of three weeks the pain and febrile disturbance left him, and the tumefaction was reduced considerably; but the scrotum remained larger than in health, was thickened, and doughy to the finger. In this state he returned to his ordinary occupations, and took a good deal of exercise. Little change was observed till the expiration of two years, when the parts became larger, had an œdematous appearance, and became gradually studded with soft smooth shining tubercles, in general appearance resembling small grapes. He had no pain, but suffered inconvenience from the size and weight of the tumour. In this state of matters, while walking the street, he suddenly felt a fluid running down his thighs, and, going into his house, found that one of the grape-like processes had given way, and that there was issuing from it a milky fluid, slightly streaked with a substance resembling, in appearance, chocolate. The discharge continued some hours, and, according to his own account, amounted to a quart. He had no further inconvenience for seven or eight days, when another discharge of similar kind and quantity took place. In this way he went on upwards of two years. Once a week, on an average, the discharge recurred, varying in quantity, but generally amounting to a pint, and retaining the appearance of milk, streaked with chocolate. During the whole period his appetite, digestion, and general health were good, and he rather gained than lost flesh. Eight months ago the scrotum was scarified superficially at a number of points, whence the said fluid issued copiously; he afterwards employed some preparation of iodine with friction to the parts, and took a course of calomel, with cicuta, till it induced salivation. Since the use of these remedies there has been less tumefaction, and little discharge, except when the parts are punctured.

Such is his own account, confirmed to a certain extent, and no where contradicted, by medical testimony.

At present the scrotal tumour is as large as a child's head of ordinary dimensions, at two years of age. On the left side of the raphe, which is tense, thick, and cord-like, it is soft, smooth, and equal, and has a sarcomatous feel. On the right side, at the upper anterior part, it is more prominent than the left; is irregular on the surface, and feels boggy—the colour being healthy, as it is over the whole surface. At this place, within short distances of each other, there are four small nipple-like processes, from which he can at any time produce the fluid in question. A few days ago, for my satisfaction, he touched two of them with a lancet, when there issued from each, in stream, a fluid like milk tinged with blood, to the amount of a gill in about ten minutes. He then arrested the discharge by the application of court plaister, stating that it would continue to flow for hours, if left to itself; and it certainly showed no sign of being exhausted, or tendency to stop. It coagulated as fast as it fell into the basin, but soon afterwards separated into two parts; one, being much the largest, a white, homogeneous, milk-like fluid, and the other a reddish, being loose coagulum, resembling the crasamentum of healthy blood.

I do not offer any conjecture regarding the source, formation, or nature of this fluid, contenting myself at present by stating, that it bears considerable likeness to the milky, or serous blood drawn from the veins, in particular conditions of the body.

The subject continues healthy and robust. He has no other appearance of disease, except considerable enlargement of the femoral lymphatics, on either side, opposite the scrotal tumour, and apparently the effect of its pressure. The testicles, as far as can be ascertained through a scrotum so much thickened, are healthy. It deserves remark, that the tumour, after the discharge of the fluid, though diminished, is not diminished to an extent commensurate with the quantity of matter discharged.

For the knowledge which I possess of this case I am indebted to Dr. Williams, a resident English practitioner."

---

